# LFER Studies Evaluating Solvent Effects on an α-Chloro-and two β,β,β-Trichloro-Ethyl Chloroformate Esters

**DOI:** 10.13179/canchemtrans.2014.02.02.0093

**Published:** 2014-02-03

**Authors:** Malcolm J. D’Souza, Brandon Sandosky, Gabriel A. Fernandez-Bueno, Matthew J. McAneny, Dennis N. Kevill

**Affiliations:** 1 Department of Chemistry, Wesley College, 120 N. State Street, Dover, DE 19901-3875, USA; 2 Department of Chemistry and Biochemistry, Northern Illinois University, DeKalb, IL 60115-2862, USA

**Keywords:** Solvolysis, Addition-Elimination, Grunwald-Winstein Equation, Ionization, 2,2,2-Trichloroethyl Chloroformate, 2,2,2-Trichloro-1,1-Dimethylethyl Chloroformate, 1-Chloroethyl Chloroformate, Phenyl Chloroformate, Linear Free Energy Relationships (LFERs)

## Abstract

To provide insight and to identify the occurrence of mechanistic changes in relation to variance in solvent-type, the solvent effects on the rates of solvolysis of three substrates, 2,2,2-trichloro-1,1-dimethylethyl chloroformate, 2,2,2-trichloroethyl chloroformate, and 1-chloroethyl chloroformate, are analyzed using linear free energy relationships (LFERs) such as the extended Grunwald-Winstein equation, and a similarity-based LFER model approach that is based on the solvolysis of phenyl chloroformate. At 25.0 °C, in four common solvents, the α-chloroethyl chloroformate was found to react considerably faster than the two β,β,β-trichloro-substituted analogs. This immense rate enhancement can be directly related to the proximity of the electron-withdrawing α-chlorine atom to the carbonyl carbon reaction center. In the thirteen solvents studied, 1-chloroethyl chloroformate was found to strictly follow a carbonyl addition process, with the addition-step being rate-determining. For the two β,β,β-trichloro-substrates, in aqueous mixtures that are very rich in a fluoroalcohol component, there is compelling evidence for the occurrence of side-by-side addition-elimination and ionization mechanisms, with the ionization pathway being predominant. The presence of the two methyl groups on the α-carbon of 2,2,2-trichloro-1,1-dimethylethyl chloroformate has additive steric and stereoelectronic implications, causing its rate of reaction to be significantly slower than that of 2,2,2-trichloroethyl chloroformate.

## 1. INTRODUCTION

Chloroformates are synthetically useful carboxylic acid esters whose chemistry [1–3] acquiesces them to have wide ranging applications as solvents, or industrial precursors, in myriad agricultural and pharmaceutical manufacturing processes [4–7]. Moreover the presence of *syn* geometry [8,9] in their structure, induces efficient chemoselective methods for cleaving and/or removing protecting groups [6,10–12]. For alkyl chloroformates, the aqueous binary solvolytic displacement behavior at the electrophilic carbonyl carbon was shown to be directly linked to both the type of alkyl group present, and to the dielectric constant of the participating solvents [13–34]. Conclusions for the majority of such solvolytic studies [19–24, 26–34], were obtained through detailed analyses procured when experimental kinetic rate data were incorporated into linear free energy relationships (LFERs), such as the extended Grunwald-Winstein (G-W) equation (equation 1) [35].

(1) $$\text{log}(k/k_{o})=lN_{T}+mY_{Cl}+c$$

In equation 1, *k* and *k_o_* are the specific rates of solvolysis in a given solvent and in 80% ethanol (the standard solvent). The sensitivity to changes in solvent nucleophilicity (*N*_T_) are approximated by *l, m* represents the sensitivity to changes in the solvent ionizing power *Y*_Cl_, and *c* is a constant (residual) term. The *N_T_* scale developed for considerations of solvent nucleophilicity is based on the solvolyses of the *S*-methyldibenzothiophenium ion [36,37]. The solvent ionizing power *Y*_Cl_ scale is based on the solvolysis of 1- or 2-adamantyl derivatives [38–42]. Equation 1 can also be applied to substitutions at an acyl carbon [43].

Whenever there is the possibility of the presence of charge delocalization due to anchimeric assistance resulting from 1,2-Wagner-Meerwein-type migrations or when, conjugated π-electrons are adjacent to the developing carbocationic center, an additional *hI* term [26,34,44–46] is added to the shown as equation 1, to give equation 2. In equation 2, *h* represents the sensitivity of solvolyses to changes in the aromatic ring parameter *I* [44–46].

(2) $$\text{log}(k/k_{o})=lN+mY+hI+c$$

**Scheme 1:**
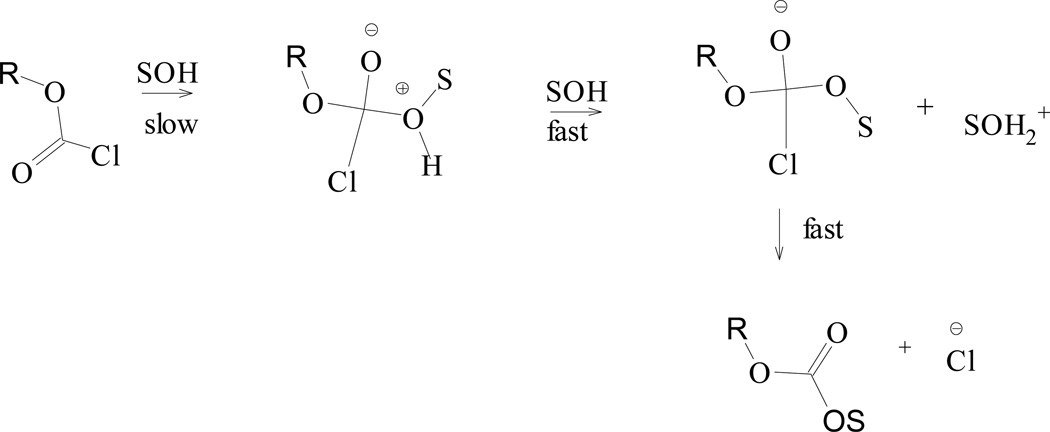
A carbonyl addition process for chloroformate esters

In a recent review chapter [34], we discuss in detail, the equations 1 and 2 analyses obtained for several examples of alkyl, aryl, alkenyl, and alkynyl chloroformate solvolyses. All of the considerations [34] indicated the immense usefulness of equations 1 and 2. We have strongly suggested [26,34,43,47] that the *l* (1.66) and *m* (0.56) values (*l/m* ratio of 2.96) obtained for the solvolysis of phenyl chloroformate (PhOCOCl, **1**) in the 49 solvents studied, be used as a standard indicator for chloroformate solvolysis pathways that incorporate a rate-determining formation of the tetrahedral intermediate in a carbonyl addition process (Scheme 1).

Substituting both oxygen atoms in **1** with sulfur, yields the dithioester phenyl chlorodithioformate (PhSCSCl, **2**). Application of equations 1 and 2 to solvolytic rate data for **2** results in *l* values of 0.69 and 0.80, and *m* values of 0.95 and 1.02 [47,48], respectively. The *l/m* ratios (0.73 and 0.78) can be considered [26,33] as good indicators for ionization (S_N_1 type) mechanisms with significant solvation at the developing thioacylium ion. (or acylium ion in the case of the chloroformate analog) The accompanying *h* value of 0.42 obtained [47,48] for **2** (using equation 2), suggests that there is a minimal charge delocalization into the aromatic ring.

**Scheme 2:**
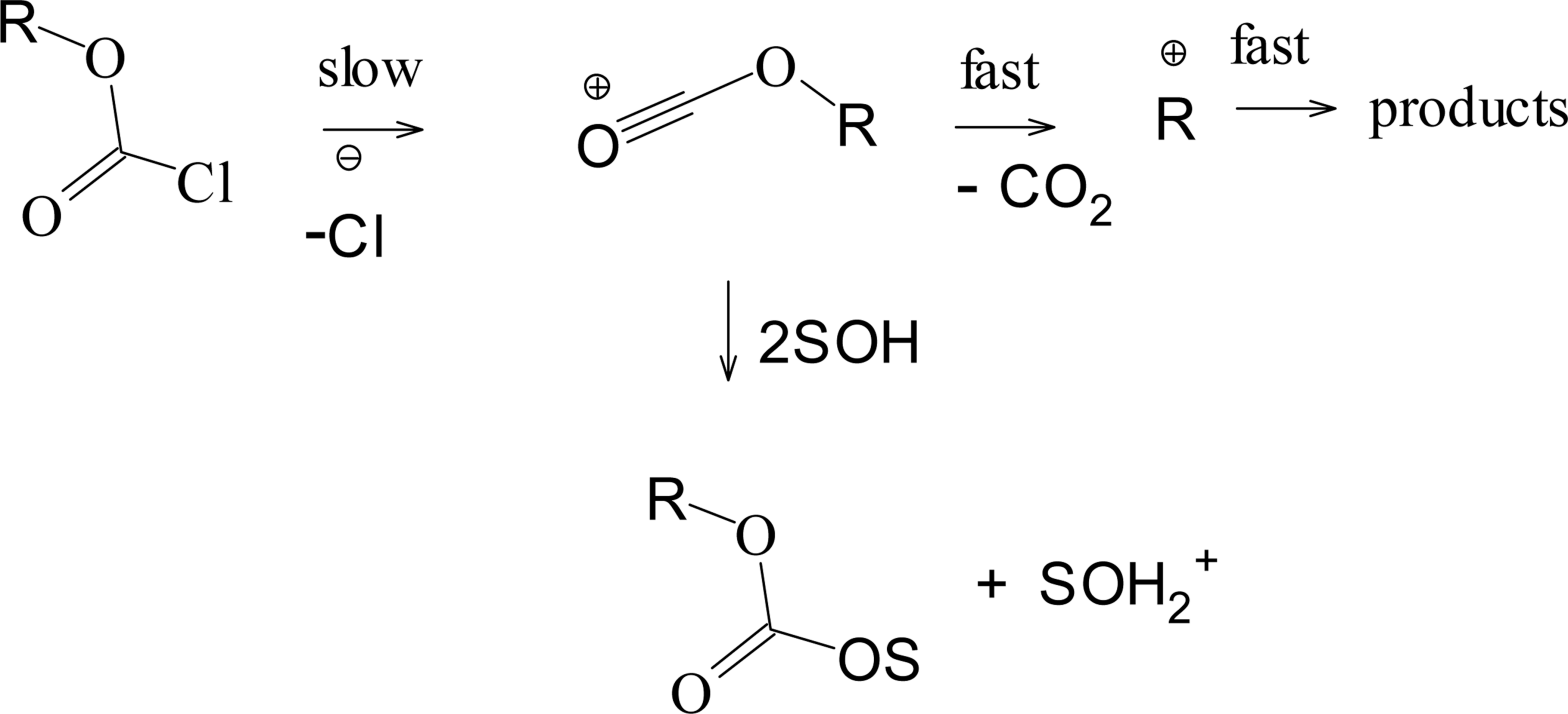
A possible unimolecular solvolytic pathway for chloroformate esters

Scheme 2 depicts a simple probable ionization with the formation of an acyl cation. There is justifiable evidence [19,23,26,27,29,34] for a concerted solvolysis-decomposition process occurring, such that the cation involved in product formation is the alkyl cation.

Likewise, several groups [9,16,17,25,28,32] have used kinetic solvent isotope effect (KSIE) studies to further probe the pseudo-first-order kinetic mechanisms of chloroformates and have provided very strong evidence, that the solvolysis of these substrates does include some general-base assistance (as indicated in Scheme 1). Our recent 2013 review chapter [34] documented the many methodical solvolytic investigations completed (to date) for structurally diverse alkyl, aryl, alkenyl, and alkynyl chloroformates. We showed that their solvolytic behavior varied between concurrent bimolecular addition-elimination (A-E) and unimolecular (S_N_1 type) ionization (or solvolysis-decomposition) pathways. The dominance of one pathway over the other was shown to be very strongly dependent on type of substrate employed, and on the solvent’s nucleophilicity and ionizing power ability [34 and references therein].

Common marketable β,β,β-trichloroalkyl chloroformates are, 2,2,2-trichloro-1-1-dimethylethyl chloroformate (**3**), and 2,2,2-trichloro-1-1-dimethylethyl chloroformate (**4**). A readily available and widely used α-chloro substituted chloroformate, is 1-chloroethyl chloroformate (**5**). All three compounds have substantial commercial use in peptide synthesis containing secondary and tertiary amines [49,50], as the carbamates developed for protection using these base-labile protection groups are easily cleaved by solvolysis [51].

Koh and Kang [28,32] followed the course of the solvolysis reactions in **3** and **4**, by measuring the change in conductivity that occurred during the reaction. They used equation 1, to analyze the kinetic rate data for **3** and **4** and obtained *l* values of 1.42 and 1.34, and *m* values of 0.39 and 0.50 in 33 and 34 different mixed solvents respectively. Additionally, they obtained relatively large kinetic solvent isotope effects (*k*_MeOH_/*k*_MeOD_) of 2.14 and 2.39. Based on these experimental results, Koh and Kang [28,32] proposed a bimolecular S_N_2 mechanism for the two β,β,β-trichloroethyl chloroformate substrates (**3** and **4**). They stipulated that the mechanism had a transition-state (TS) where the bond-making component was more progressed, and based on their experimental *k*_MeOH_/*k*_MeOD_ values, suggested that this S_N_2 TS is assisted by general-base catalysis. When the report of the Koh and Kang study of **3** appeared [28], the Wesley College undergraduate research group was independently following the rates of its reaction using a titrimetric method of analysis [52].

## 2. EXPERIMENTAL METHODS

**Figure 1:**
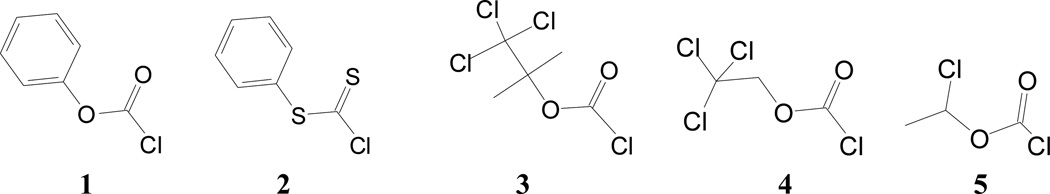
Molecular structures of phenyl chloroformate (**1**), phenyl chlorodithioformate (**2**), 2,2,2-trichloro-1,1-dimethylethyl chloroformate (**3**), 2,2,2-trichloroethyl chloroformate (**4**), and 1-chloroethyl chloroformate (**5**)

The 2,2,2-trichloro-1,1-dimethylethyl chloroformate (**3**, 96%, Sigma-Aldrich) and the 1-chloroethyl chloroformate (**5**, 98%, Sigma-Aldrich) were used as received. Solvents were purified as described previously [20]. For **3** and **5**, a substrate concentration in the 0.003 – 0.009 M range in a variety of solvents was employed. For **3**, the 25.0 mL binary solution mixtures were first allowed to equilibrate in a 35.0 °C constant-temperature water bath and then, the progress of the reaction was monitored by titrating aliquots of the solution using a lacmoid indicator. The rapid kinetic runs of **5** were followed using a conductivity cell containing 15 mL of solvent which was first allowed to equilibrate in a 25.0 °C constant-temperature water bath, with stirring. The specific rates and associated standard deviations, as presented in Table 1, are obtained by averaging all of the values from, at least, duplicate runs. Multiple regression analyses were carried out using the Excel 2007 package from the Microsoft Corporation. The molecular structures (*syn* geometry) presented in Figure 1, were drawn using the KnowItAll^®^ Informatics System, ADME/Tox Edition, from BioRad Laboratories, Philadelphia, PA.

## 3. RESULTS AND DISCUSSIONS

In Table 1, we report the pseudo first order rate coefficients obtained for **3** at 35.0 °C, and for **5** at 25.0 °C, in 19 and 13 diverse binary aqueous organic solvents, respectively. Also presented in Table 1, are the *N*_T_ and *Y*_Cl_ values that are needed in equation 1 to compute the necessary bond-making (*l* value), bond-breaking (*m* value), and residual (*c* value) components.

The data in Table 1 shows that the specific rates of solvolysis of 2,2,2-trichloro-1,1-dimethylethyl chloroformate (**3**) gradually increases with the increase in water-content in ethanol (EtOH), methanol (MeOH), acetone, 2,2,2-trifluoroethanol (TFE), and 1,1,1,3,3,3-hexafluro-2-propanol (HFIP) mixtures. In the pure organic mixtures of 2,2,2-trifluoroethanol and ethanol (T-E), the rate increases with an increase in ethanol content. These broad observations on the solvent influences of the rate constants for **3** suggest that the solvent nucleophilic component (sensitivity indicated by *l* value) plays an important role in rate-determining step of the reaction. The experimental values of our rate determinations (for **3**) are within an acceptable 2–10% range when compared to those obtained by Koh and Kang [28] in 60T-40E, and in the aqueous mixtures of ethanol, methanol, acetone, and TFE. However, in ethanol, methanol, 20T-80E and 70% HFIP at 35.0 °C, our results differed from the Koh and Kang [28] values by 15%, 18%, 33% and 61% respectively. In these four solvents, the rate data that we report in Table 1 are the average specific rates obtained after 4–8 different independent determinations; using different batches of solvents and containing multiple samples of varying concentrations of **3** that were purchased at different times. It is of utmost interest that the most significant deviations have occurred in solvents where sensitivity to general-base catalysis is the greatest. This is due to the solvents hydrogen-bond donating ability (typically in the order of HFIP > TFE > MeOH > water > ethanol) being a factor in the stabilizing of the developing transition-state [54].

For 1-chloroethyl chloroformate (**5**), the specific rate increase is much more pronounced with increases in the solvents nucleophilic ability (*N*_T_ value). In the strongly hydrogen-bonding fluoroalcohols, we obtained rates in three aqueous TFE solutions and two TFE-EtOH mixtures, but were unable to obtain reliable and repeatable rates in the highly ionizing HFIP mixtures.

In Table 2, we list the specific rates of reaction for the previously examined primary and secondary alkyl chloroformates that follow similar mechanistic patterns in five common solvents at 25.0 °C. Included are methyl chloroformate (MeOCOCl) [21], ethyl chloroformate (EtOCOCl) [20], 2,2,2-trichloroethyl chloroformate (**4**) [32], *n*-propyl chloroformate (*n*-PrOCOCl) [24], *iso*-propyl chloroformate (*i*-PrOCOCl) [27], iso-butyl chloroformate (*i*-BuOCOCl) [30], and *n*-octyl chloroformate (*n*-OctOCOCl) [53]. Data for **3** and **5** are also shown in Table 2.

The 1-adamantyl and 2-adamantyl chloroformate (1-AdOCOCl and 2-AdOCOCl) [19,23] favor a solvolysis-decomposition type pathway in a majority of the solvents studied, and neopentyl chloroformate (neoPOCOCl) [29], whose mechanism parallels those listed in the non-fluoroalcohol mixtures, was studied at 45.0°C. Concurrent addition-elimination (A-E) and ionization mechansims were proposed for ethyl chloroformate (EtOCOCl) [20], with the ionization (S_N_1-type) pathway being favored in the highly ionizing fluoroalcohol mixtures. Additionally for the secondary chloroformate, *i*-PrOCOCl, a solvent-decomposition mechanism was shown to dominate in 70 TFE [27].

In MeOH, EtOH, and 80% EtOH, there is a 10 to 1000-fold increase in the rates of reaction with the introduction of chlorine at the α- or β-carbon of the primary alkyl chloroformate esters. This tendency for such compelling rate increases results from the inductive effects that are introduced due to the presence of electron-withdrawing chlorine (as substituents) on the primary alkyl chain.

For **3, 4**, and **5**, in the pure and aqueous alcohols, we observe the general progression of *k***_5_** ≫ *k***_4_** > *k***_3_**. Such forceful advancements can only develop from the immense strength of the inductive effect present in **5**, mainly due to the proximity of the electron withdrawing α-chloro substituent to the electrophilic reaction center. The *k***_3_** < *k***_4_** observations are due the additive steric and stereoelectronic effects introduced by the two methyl substituents on the α-carbon atom in **3**.

In Table 3, we list the Grunwald-Winstein parameters obtained from the literature, for PhOCOCl [43,47], and the other pertinent alkyl chloroformates that are mentioned in this research article.

In order to interpret detailed mechanisms of reaction for **3, 4**, and **5**, we have also reanalyzed and documented the resultant multiple regression values that were obtained on using equation 1. For use as a mechanistic criterion, we also considered the *l/m* ratios of the cataloged chloroformate substrates, since it was convincingly shown [53] that *n*-octyl fluoroformate, which has an *l/m* ratio of 2.28, proceeds by a rate-determining carbonyl-addition (A-E) process. This assignment was supported by the observation that in a number of common solvents the *k*_F_/*k*_Cl_ ratios for *n*-octyl fluroformate and *n*-octyl chloroformate was greater than unity [53].

Our solvolysis study for 2,2,2-trichloro-1,1-dimethylethyl chloroformate (**3**) at 35.0 °C, included 19 solvents that had very widely varying ranges of solvent nucleophilicity and solvent ionizing power. Analyses (using equation 1) of the rates obtained for **3** in these solvents resulted in an *l* value of 1.17 ± 0.17, an *m* value of 0.29 ± 0.13, a *c* value of 0.03 ± 0.16, an *F*-test value of 67, and a multiple correlation coefficient (*R*) value of 0.945. These *l* an *m* values are on the much lower side of the spectrum when compared to those tabulated in Table 3 for the previously studied alkyl chloroformate esters. Furthermore, the *m* value obtained for **3** has a *P* value (probability of statistical significance) of 0.03. Using literature values [43,47] for PhOCOCl, in the same 19 solvents (and using an interpolated rate of 1.49 × 10^−4^s^−1^ for PhOCOCl in 70T-30E), we obtained, 1.62 ± 0.11, 0.54 ± 0.08, 0.24 ± 0.11, 229, and 0.983, for *l, m, c, F*-test, and *R*, respectively.

**Figure 2:**
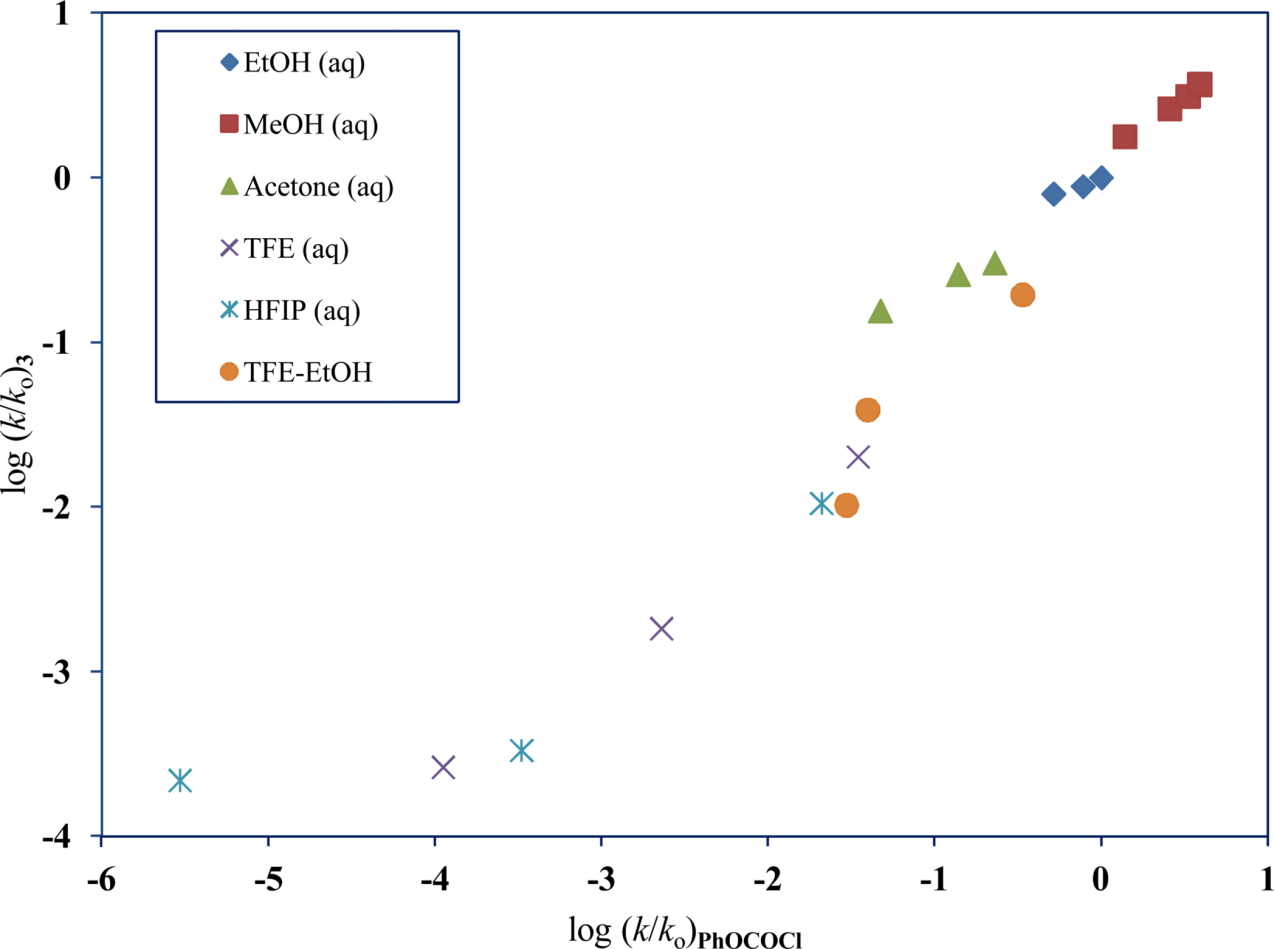
The plot of log (*k/k_o_*)**_3_** against log (*k/k*_o_)**_PhOCOCl_**

A plot of the log (*k/k*_o_)**_3_** versus log (*k/k*_o_)**_PhOCOCl_** is shown in Figure 2. This graph has a slope of 0.831 ± 0.058, an intercept of −0.099 ± 0.118, an *F*-test value of 204, and a best-fit linear regression (r^2^) value of 0.961. The Figure 2 residual plot clearly shows that the 97 HFIP point deviates significantly from the best-fit line. Removal of this 97 HFIP value results in a slope of 0.988 ± 0.002, an intercept of −0.002 ± 0.073, an improved *F*-test value of 483, and an enhanced r^2^ value of 0.984. Such improvements strongly illustrate that for **3**, a similar PhOCOCl addition-elimination (A-E) type mechanism (Scheme 1) occurs in the remaining 18 solvents.

On omitting the 97 HFIP rate value for **3** and reanalyzing the remaining 18 solvents (Table 1) with equation 1, we obtain an *l* value of 1.43 ± 0.15, an *m* value of 0.38 ± 0.10 (associated *P* value = 0.002), a *c* value of 0.17 ± 0.13, *F*-test = 96, and *R* = 0.963 (reported in Table 3). Here, **3** has an *l/m* ratio of 3.76. In the identical 18 solvents studied, a reanalysis (with equation1) for PhOCOCl leads to values of 1.61 ± 0.13, 0.53 ± 0.09, and 0.23 ± 0.12, for *l, m*, and *c*, respectively. The *l/m* ratio for PhOCOCl is 3.04. These robust *l* and *m* values obtained for PhOCOCl, have an associated *F*-test value of 127 and *R* = 0.972. The larger *l/m* ratio for **3** indicates that it is more susceptible (when compared to PhOCOCl) to general-base catalysis.

**Figure 3:**
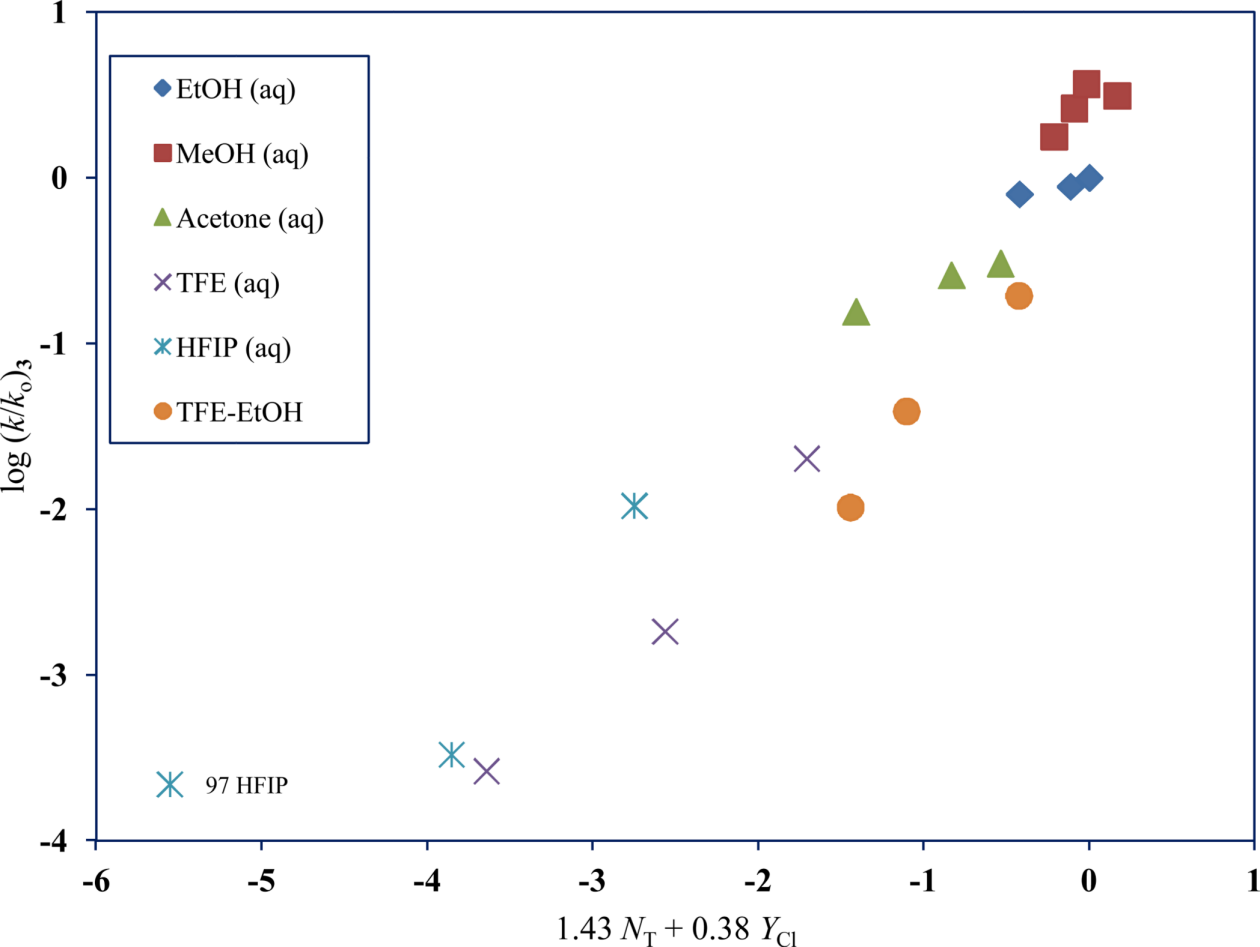
The plot of log (*k/k_o_*) for 2,2,2-trichloro-1,1-dimethylethyl chlorothioformate (**3**) against 1.43 *N*_T_ + 0.38 *Y*_Cl_ in nineteen pure and binary solvents. The 97 HFIP point was not included in the correlation. It is added to the plot to show the extent of its deviation

A plot of log (*k/k_o_*) for 2,2,2-trichloro-1,1-dimethylethyl chloroformate (**3**) against 1.43 *N*_T_ + 0.38 *Y*_Cl_ is shown in Figure 3 with the deviation for the 97 HFIP point indicated. Using log (*k/k_o_*)**_3_** = 1.43 *N*_T_ + 0.38 *Y*_Cl_ + 0.17, we calculated an expected bimolecular carbonyl-addition rate for **3** to be 3.35 × 10^−9^ in 97 HFIP. Comparing this calculated value to the experimental value obtained for **3** in 97 HFIP (and shown in Table 1), we can definitively conclude that in this highly ionizing mixture, the mechanism of reaction is of the S_N_1 type, with 98% of reaction following the ionization pathway.

Koh and Kang [32] measured the rate constants for solvolyses of 2,2,2-trichloroethyl chloroformate (**4**) in 34 pure and binary solvent mixtures at 35.0 °C. Using their data [32], we reanalyzed the reported rates of reaction using equation 1 and obtained, *l* = 1.35 **±** 0.07, *m* = 0.51 **±** 0.04, *c* = 0.07 ± 0.06, *F*-test = 175, and *R* = 0.958. Our *l* and *m* values match the ones reported [32] for **4**. The *l/m* ratio for **4** works out to be 2.65. Analyzing the literature data for PhOCOCl [43,47] in the identical 34 solvents, we obtain, *l* = 1.52 **±** 0.08, *m* = 0.52 **±** 0.04, *c* = 0.11 ± 0.07, *F*-test = 188, and *R* = 0.961 (*l/m* ratio = 2.92).

**Figure 4:**
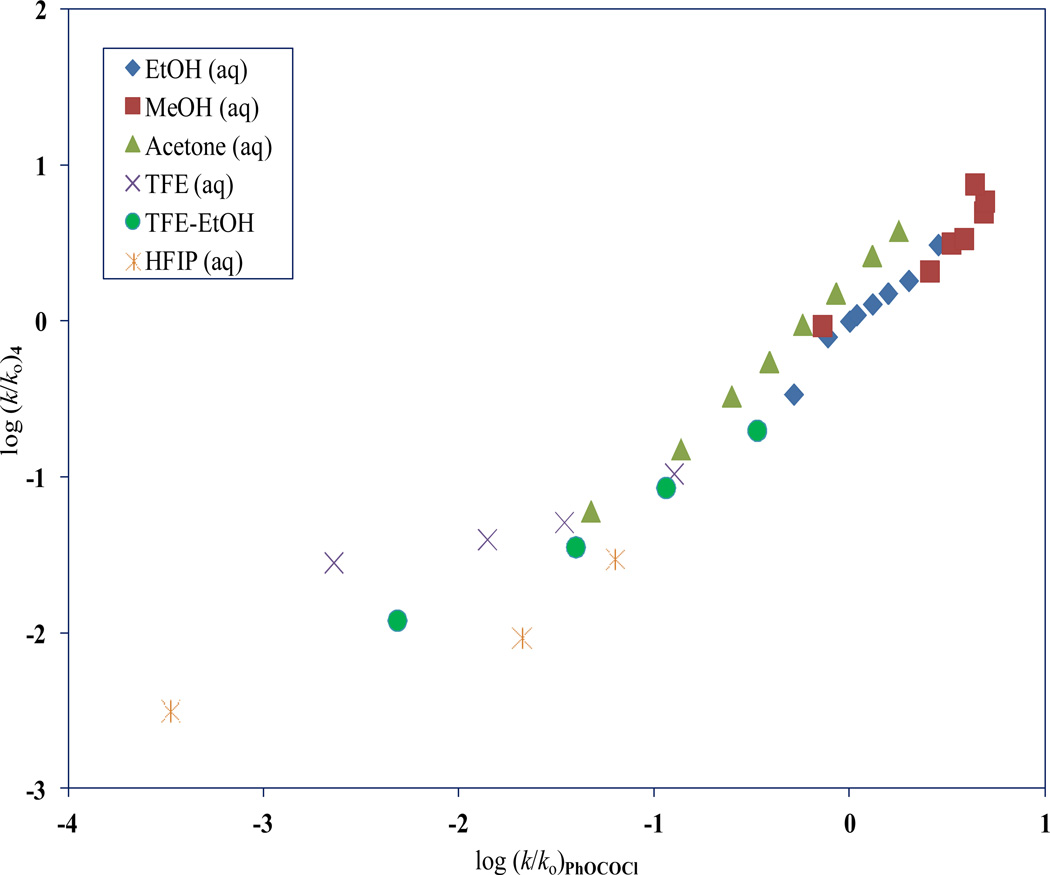
The plot of log (*k/k_o_*)**_4_** against log (*k/k*_o_)**_PhOCOCl_**

A plot of log (*k/k*_o_)**_4_** versus log (*k/k*_o_)**_PhOCOCl_** is shown in Figure 4. This plot has a slope = 0.85 ± 0.04, *c* = 0.03 ± 0.05, *F*-test = 374, and r^*2*^ = 0.960. A visual inspection of the scatter plot (Figure 4) reveals that the 90 HFIP and 90 TFE points are markedly dispersed. The removal of these two points increases the *F*-test value to 554 and the r^2^ value rises to 0.974. The slope is now 0.99 ± 0.04, and *c* = 0.04 ± 0.04. The improved r^2^ value hints that the two substrates (**4** and PhOCOCl) proceed via similar mechanisms in the remaining 32 solvents.

An analysis (Table 3) using equation 1 for **4** in the remaining 32 solvents yields, *l* = 1.52 ± 0.08, *m* = 0.55 ± 0.03, *c* = 0.01 ± 0.06, *F*-test = 178, and *R* = 0.962. In corresponding solvents for PhOCOCl, an analysis using equation 1, produces *l* = 1.47 ± 0.10, *m* = 0.51 ± 0.04, *c* = 0.10 ± 0.07, *F*-test = 105, and *R* = 0.938. The *l/m* ratio for **4** is 2.76 and that for PhOCOCl is 2.88, thus illustrating that solvolyses of both **4** and PhOCOCl proceed through very similar carbonyl-addition tetrahedral transition-state.

Using log (*k/k*_o_)**_4_** = 1.52*N*_T_ + 0.55*Y*_Cl_ + 0.01, we calculated the expected bimolecular carbonyl-addition (A-E) rates for 90 HFIP and 90 TFE to be 4.90 × 10^−6^ s^−1^ and 6.19 × 10^−5^ s^−1^. Comparing these calculated rates to the ones that were experimentally determined in 90 HFIP and 90 TFE [32], we project that the ionization (S_N_1) component for **4** in these two solvents are, 87% and 82% respectively.

**Figure 5:**
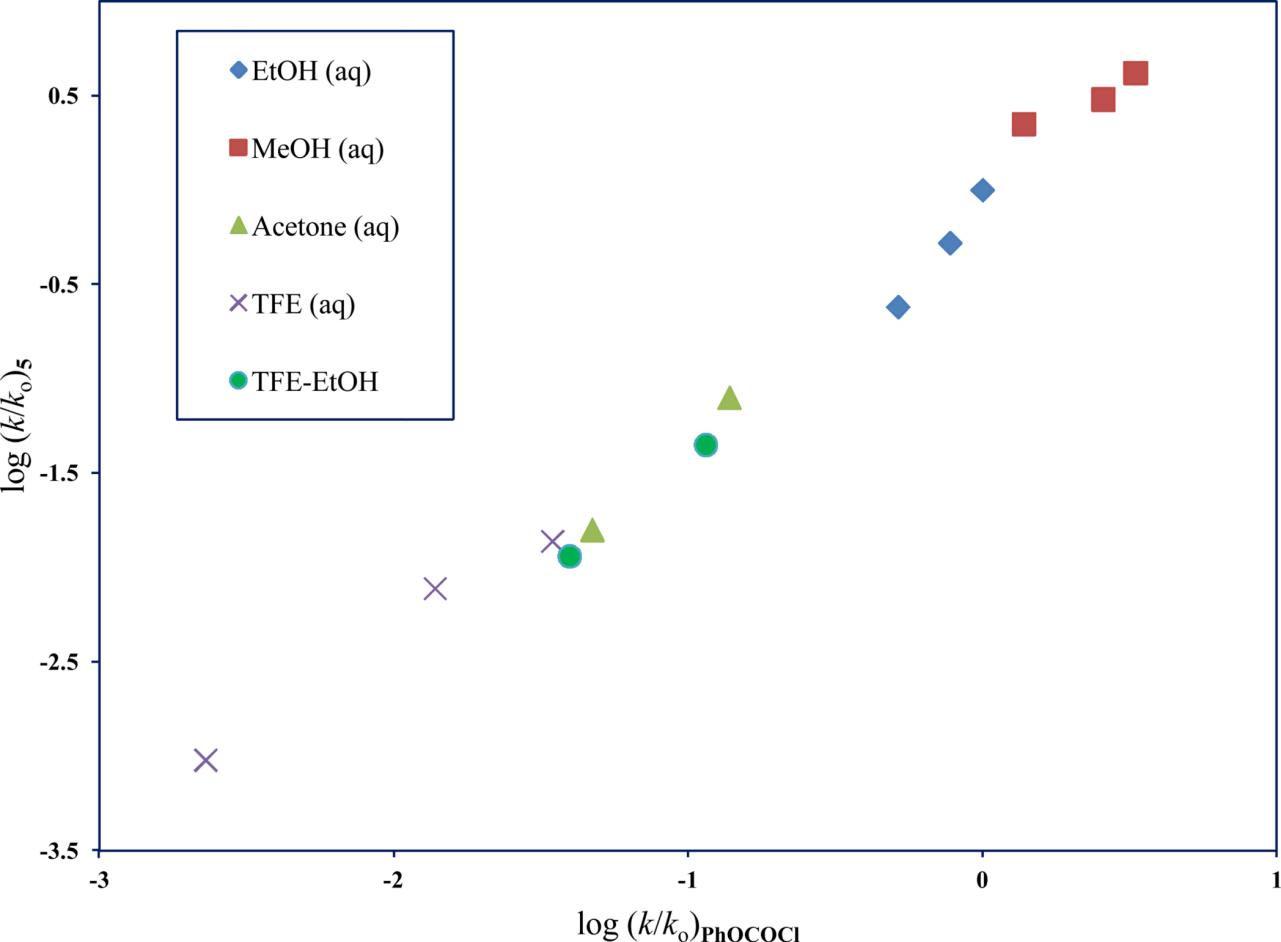
The plot of log (*k/k_o_*)**_5_** against log (*k/k*_o_)**_PhOCOCl_**

Due to a variety of experimental difficulties we could only study the solvolysis of the monochloro substrate, 1-chloroethyl chloroformate (**5**), in 13 pure and aqueous binary mixtures at 25.0 °C. A plot of log (*k/k*_o_)**_5_** against log (*k/k*_o_)**_PhOCOCl_** is shown in Figure 5. This plot has a slope of 1.19 ± 0.05, an intercept of −0.07 ± 0.06, an *F*-test value of 603, and an r^2^ value of 0.991. The considerable *F*-test value accompanied by an excellent r^2^ value, indicates that this is indeed a well-fitting regression model, and that the two substrates (PhOCOCl and **5**) have very similar transition-state character. The slightly greater than unity slope further suggests that **5** has a slightly later transition-state (as compared to PhOCOCl).

For **5** an analysis using equation 1 of solvolyses rates in all of the thirteen solvents studied, results in *l* = 1.99 ± 0.23, *m* = 0.62 ± 0.12, *c* = 0.19 ± 0.17, *F*-test = 49, and *R* = 0.953. The *l/m* ratio is 3.21 for **5**. In the identical thirteen solvents, an equation 1 analysis for PhOCOCl yields, *l* = 1.61 ± 0.15, *m* = 0.47 ± 0.08, *c* = 0.19 ± 0.11, *F*-test = 90, *R* = 0.973, and the *l/m* ratio = 3.42. A comparison of the *l/m* ratios for these two substrates again illustrates the similarities in the tetrahedral addition-elimination transition-states.

## 4. CONCLUSION

The interplay between electronic and steric effects amongst the three chloro-substituted chloroformates studied, is clearly evident in the rate order *k***_5_** ≫ *k***_4_** > *k***_3_** observed. The α-chloro-substituent in 1-chloroethyl chloroformate (**5**) exerts very large electron-withdrawing inductive effects and, as a result, it leads to rates of reaction that are orders of magnitude higher. The presence of the electron-withdrawing trichloromethyl group in 2,2,2-trichloroethyl chloroformate (**4**) also plays an advantageous role in accelerating the addition step of an addition-elimination reaction, whereas the comparatively sterically encumbered 2,2,2-trichloro-1,1-dimethylethyl chloroformate (**3**), had the lowest rates that were influenced by counteractive electronic and steric effects.

Coupling theories of linear-free energy relationships (LFERs) that employ a similarity-model approach based on the solvolysis of phenyl chloroformate (**1**), together with the information derived from the extended Grunwald-Winstein (equation 1) analysis, present a consistent picture for the solvolysis mechanisms of **3, 4**, and **5**.

A log (*k/k*_o_) plot of **3** against **1**, reveals a large-scale divergence for the 97 HFIP point. Neglecting this 97 HFIP data point for **3** in the Grunwald-Winstein computation, led to an *l/m* ratio of 3.76, which is solidly indicative of a carbonyl-addition process that is assisted by general-base catalysis. This also indicates that the ionization pathway is the dominant process (98%) for **3** in 97 HFIP.

Utilizing the previously published rates, a log (*k/k*_o_) plot of **4** against **1**, displayed some disparity in the 90 HFIP and 90 TFE values. On their removal and then applying the equation 1 to the rates in the remaining 32 solvents, we acquired an *l/m* ratio of 2.76 for **4**, which was found to be very close to the 2.88 value for **1** in identical solvents. This supports our proposal that the tetrahedral carbonyl-addition transition-state **4** is analogous to that of **1**.

The log (*k/k*_o_) plot of **5** against **1** was near ideal, with an r^2^ value of 0.991, and a slope that was slightly greater than unity. The similar *l/m* ratios for **5** and **1** verified that the two substrates had virtually indistinguishable tetrahedral transition-state structure.

## Figures and Tables

**Table 1:** Specific rates of solvolysis (*k*) of **3** at 35.0 °C and **5** at 25.0 °C in several pure and binary solvents respectively. Also listed are the literature values for *N_T_* and *Y_Cl_*

Solvent (%)^a^	3; 10^4^*k,* s^−1^^b^	5; 10^4^*k*, s^−1^^b^	*N_T_*^c^	*Y_Cl_*^d^
100% EtOH	6.49 ± 0.27	54.3 ± 0.8	0.37	−2.50
90% EtOH	7.24 ± 0.10	118 ± 3	0.16	−0.90
80% EtOH	8.18 ±0.11	226 ± 5	0.00	0.00
100% MeOH	14.5 ± 0.6	509 ± 2	0.17	−1.2
90% MeOH	21.5 ± 0.1	682 ± 0	−0.01	−0.20
80% MeOH	25.5 ± 0.2	953 ± 1	−0.06	0.67
70% MeOH	30.1 ± 0.7		−0.40	1.46
90% Acetone	1.27 ± 0.07	3.62 ± 0.08	−0.35	−2.39
80% Acetone	2.11 ± 0.09	18.1 ± 0.1	−0.37	−0.83
70% Acetone	2.48 ± 0.08		−0.42	0.17
97% TFE (w/w)	0.00217 ± 0.00022		−3.30	2.83
90% TFE (w/w)	0.0150 ± 0.0009	0.215 ± 0.000	−2.55	2.85
80% TFE (w/w)		1.77 ± 0.01	−2.19	2.90
70% TFE (w/w)	0.165 ± 0.009	3.09 ± 0.01	−1.98	2.96
70T-30E	0.0839 ± 0.0021		−1.34	1.24
60T-40E	0.319 ± 0.007	2.59 ± 0.00	−0.94	0.63
40T-60E		10.2 ± 0.0	−0.34	−0.48
20T-80E	1.59 ± 0.17		0.08	−1.42
97% HFIP (w/w)	0.00178 ± 0.00023		−5.26	5.17
90% HFIP (w/w)	0.00273 ± 0.00021		−3.84	4.41
70% HFIP (w/w)	0.0858 ± 0.0024		−2.94	3.83

a Substrate concentration of *ca.* 0.0052 M; binary solvents on a volume-volume basis at 25.0 °C, except for TFE-H_2_O and HFIP-H_2_O solvents which are on a weight-weight basis. T-E are TFE-ethanol mixtures.

b With associated standard deviation.

c Ref [36, 37].

d Ref [38–41].

**Table 2:** A comparison of the specific rates of solvolysis (10^5^*k*, s^−1^) of methyl chloroformate (MeOCOCl) [21], ethyl chloroformte (EtOCOCl) [20], **3** [28], **4** [32], **5**, *n*-propyl chloroformate (*n*-PrOCOCl) [24], *iso*-propyl chloroformate (*i*-PrOCOCl) [22,27], *iso*-butyl chloroformate (*i*-BuOCOCl) [30], and *n*-octyl chloroformate (*n*-OctOCOCl) [53] in common solvents at 25.0 °C

Solvent (%)	MeOCOCl	EtOCOCl	3	4	5	*n*-PrOCOCl	*i*-PrOCOCl	*i*-BuOCOCl	*n*-OctOCOCl
MeOH	15.6	8.24	85.7	605	5093	8.88	4.19	9.89	8.51
EtOH	3.51	2.26	25.8	231	543	2.20	1.09	2.36	2.39
80EtOH	17.2	7.31	42.0	711	2264	7.92	3.92	8.17	7.37
97TFE		0.023				0.062	12.3	0.086	
70TFE	0.857	0.611	0.838	3.29	30.9	0.591	19.7	0.481	

**Table 3:** Correlation of the specific rates of solvolysis of **3, 4**, and **5** (this study) and several other chloroformate esters (values from the literature), using the extended Grunwald-Winstein equation (equation 1)

Substrate	*n*^a^	*l*^b^	*m*^b^	*c*^b^	*l/m*	*R*^c^	*F*^d^	*Mechanism*
PhOCOCl^e^	49	1.66 ± 0.05	0.56 ± 0.03	0.15 ± 0.07	2.95	0.980	568	A-E^f^
2-AdOCOCl^e^	19	0.03 ± 0.07	0.48 ± 0.04	−0.10 ± 0.09	0.06	0.971	130	I^g^
1-AdOCOCl^e^	11	0.08 ± 0.20	0.59 ± 0.05	0.06 ± 0.08	0.14	0.985	133	I^g^
MeOCOCl^e^	19	1.59 ± 0.09	0.58 ± 0.05	0.16 ± 0.07	2.74	0.977	171	A-E
EtOCOCl^e^	28	1.56 ± 0.09	0.55 ± 0.03	0.19 ± 0.24	2.84	0.967	179	A-E
	7	0.69 ± 0.13	0.82 ± 0.16	−2.40 ± 0.27	0.84	0.946	17	S_N_1
*n*-PrOCOCl^e^	22	1.57 ± 0.12	0.56 ± 0.06	0.15 ± 0.08	2.79	0.947	83	A-E
	6	0.40 ± 0.12	0.64 ± 0.13	−2.45 ± 0.27	0.63	0.942	11	S_N_1
*i*-PrOCOCl^e^	9	1.35 ± 0.22	0.40 ± 0.05	0.18 ± 0.07	3.38	0.960	35	A-E
	16	0.28 ± 0.04	0.59 ± 0.04	−0.32 ± 0.06	0.47	0.982	176	I^g^
*i*-BuOCOCl^e^	18	1.82 ± 0.15	0.53 ± 0.05	0.18 ± 0.07	3.43	0.957	82	A-E
neoPOCOCl^e^	13	1.76 ± 0.14	0.48 ± 0.06	0.14 ± 0.08	3.67	0.977	226	A-E
	8	0.36 ± 0.10	0.81 ± 0.14	−2.79 ± 0.33	0.44	0.938	18	S_N_1
PhSCSCl^e^	31	0.69 ± 0.05	0.95 ± 0.03	0.18 ± 0.05	0.72	0.987	521	S_N_1
3	18^h^	1.43 ± 0.15	0.38 ± 0.10	0.17 ± 0.13	3.76	0.963	96	A-E
4	32^i^	1.52 ± 0.08	0.55 ± 0.03	0.13 ± 0.06	2.76	0.962	178	A-E
5	13	1.99 ± 0.23	0.62 ± 0.12	0.19 ± 0.17	3.21	0.953	49	A-E

a *n* is the number of solvents.

b With associated standard error.

c Multiple Correlation Coefficient.

d *F*-test value.

e See text for references giving the source of this data.

f Addition-elimination.

g Ionization-fragmentation.

h No 97 HFIP.

i No 90 HFIP, 90 TFE.
